# A comparative analysis of divergently-paired genes (DPGs) among *Drosophila *and vertebrate genomes

**DOI:** 10.1186/1471-2148-9-55

**Published:** 2009-03-11

**Authors:** Liang Yang, Jun Yu

**Affiliations:** 1 James D. Watson Institute of Genome Sciences, College of Life Sciences, Zhejiang University, Hangzhou, PR China; 2 CAS Key Laboratory of Genome Sciences and Information, Beijing Institute of Genomics, Chinese Academy of Sciences, Beijing, PR China

## Abstract

**Background:**

Divergently-paired genes (DPGs) are defined as two adjacent genes that are transcribed toward the opposite direction (or from different DNA strands) and shared their transcription start sites (TSSs) less than 1,000 base pairs apart. DPGs are products of a common organizational feature among eukaryotic genes yet to be surveyed across divergent genomes over well-defined evolutionary distances since mutations in the sequence between a pair of DPGs may result in alternations in shared promoters and thus affect the function of both genes. By sharing promoters, the gene pairs take the advantage of co-regulation albeit bearing doubled mutational burdens in maintaining their normal functions.

**Results:**

*Drosophila melanogaster *has a significant fraction (31.6% of all genes) of DPGs which are remarkably conserved relative to its gene density as compared to other eukaryotes. Our survey and comparative analysis revealed different evolutionary patterns among DPGs between insect and vertebrate lineages. The conservation of DPGs in *D. melanogaster *is of significance as they are mostly housekeeping genes characterized by the absence of TATA box in their promoter sequences. The combination of Initiator and Downstream Promoter Element may play an important role in regulating DPGs in *D. melanogaster*, providing an excellent niche for studying the molecular details for transcription regulations.

**Conclusion:**

DPGs appear to have arisen independently among different evolutionary lineages, such as the insect and vertebrate lineages, and exhibit variable degrees of conservation. Such architectural organizations, including convergently-paired genes (CPGs) may associate with transcriptional regulation and have significant functional relevance.

## Background

How genes are structurally organized and functionally evolved are two fundamental biological questions to be addressed across diverse evolutionary lineages. The best known example for structurally-coordinated and functionally-related genes are operons in prokaryotes [1]. In eukaryotes, certain classes of genes are also non-randomly distributed, forming different structural classes including pairing and clustering. For instance, genes within the same metabolic pathways are often clustered together [2] and have correlated expression patterns when compared against random genes [3-7].

Recently, there have been increasing numbers of genome-wide studies on divergently-paired genes or DPGs in human [8-11] and *Drosophila melanogaster *[12]. DPGs are often defined as two adjacent genes that are divergently transcribed on opposite DNA strands, which have transcription start sites (TSSs) less than 1,000 bp apart [10]. The sequences between the two TSSs among DPGs are defined as divergently-shared promoters (DSPs). More than 10% of the human genes are arranged in the divergent organization, and DPGs are often co-ordinately expressed with evolutionary conservation and functional association [10,11].

Among species as diverse as human [13-17], mouse [18-20], chicken [21,22], fruit fly [23,24], *Saccharomyces cerevisiae*[25,26], and *Aspergillus nidulans *[27], a substantial number of individual DPGs have been reported based on experimental evidence but few genome-wide analysis across diverse evolutionary lineages has been published. The recent availability of genome sequences of *D. melanogaster *(*Dmel*) and a constellation of closely-related species at various levels of divergence time selected in the genus *Drosophila *have made the genus an ideal model for a thorough comparative analysis for DPGs .

We performed a genome-wide identification of DPGs in *Dmel *and other selected eukaryotic genomes, including representatives from vertebrate and other sequenced *Drosophila *species. We also examined the conservation of divergent gene organization over different evolutionary time scales using orthologous sequence datasets based on synonymous (*Ks*) and nonsynonymous (*Ka*) substitution rates. We also correlated gene expression and functional relevance among DPGs of *Dmel *and other eukaryotes based on Gene Ontology terms. Our results suggested that the divergent gene organization is a widespread and evolutionary conserved feature of co-regulated transcription for functionally-related genes in *Drosophila *genomes albeit variable patterns observed among different taxonomic groups or lineages in terms of structural conservation.

## Results

### Identification and characterization of DPGs in *Dmel *and other eukaryotes

**Figure 1:**
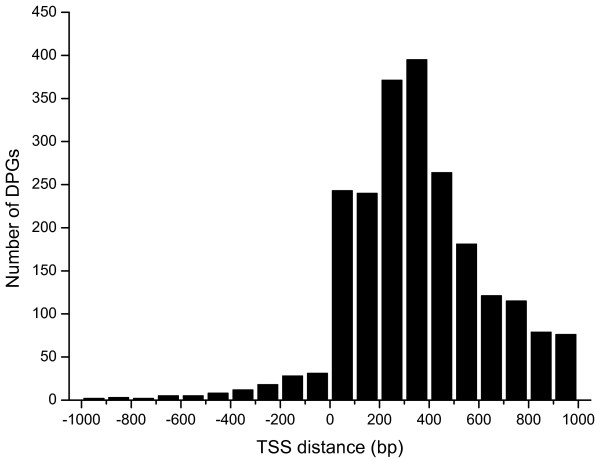
**The TSS distance of DPGs in *Dmel *genome**. The number of DPGs is plotted as a function of TSS distance between -1 kb and +1 kb.

We determined 2,199 DPGs (or 4,323 individual genes) from 13,678 annotated *Dmel *genes, accounted for 31.6% of the total [see Additional file 1]. The majority (59.0%) of the sequences between TSSs of these DPGs are less than 400 bp in length with the majority ranging from 200 to 400 bp in length (Figure 1). Since there is a possibility where a gene overlaps with two DPGs simultaneously when gene density is high enough, we also extracted multiple DPGs [see Additional file 1, the Genes in Multiple Pairs sheet]. Of the 2,199 total, only 114 pairs (5.2%) were found overlapping at the 5'ends, whereas 2,085 pairs (94.8%) are non-overlapping. In addition, we determined that 94 (4.3%) DPGs are tandem duplicates [see Additional file 1].

**Figure 2:**
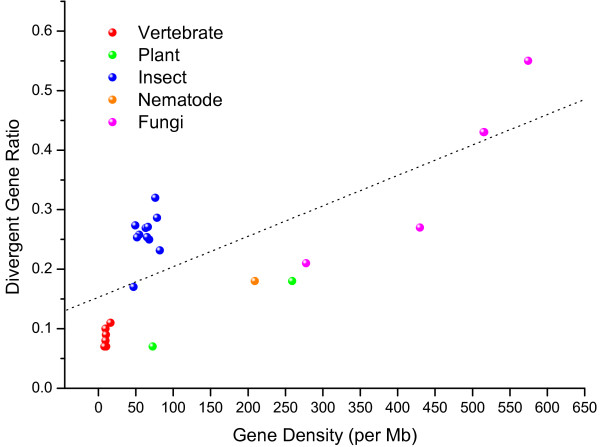
**The relationship between gene density and proportion of DPGs among eukaryotic genomes**. The divergent ratio was calculated by dividing the number of DPGs over the total number of genes in a genome. The species among various lineages including vertebrates, insects, nematodes, fungi, and plants are indicated with solid circles in red, blue, orange, magenta, and green, respectively. The dished line is added to indicate linear regression. The gene densities of insects and vertebrates are much lower than other eukaryotes and the Drosophila species have slightly higher proportions of DPGs than the vertebrates.

We also characterized DPGs among other eukaryotic genomes [see Additional file 2]. The majority of these DPGs have TSS distance s from0 to 400 bp among *Dmel *and vertebrates [see Additional file 3]. In addition, vertebrates have a relatively higher proportion of DPGs with overlapping sequences. The proportions of DPGs among other eukaryotes ranged from 6% to55% in densities so that the divergent gene organization is widespread among eukaryotic genomes. Although the relationship between gene density and the proportion of divergent genes are observed as somewhat linearly correlated (Spearman's rank correlation coefficient, *ρ *= 0.64, *p*-value = 4.3e-4) among certain lower eukaryotes, the *Drosophila *species as well as the vertebrates showed different proportions of DPGs (Figure 2); insects appear to have higher proportions as compared to those of the vertebrates [see Additional file 4].

### The evolution of DPGs

**Figure 3:**
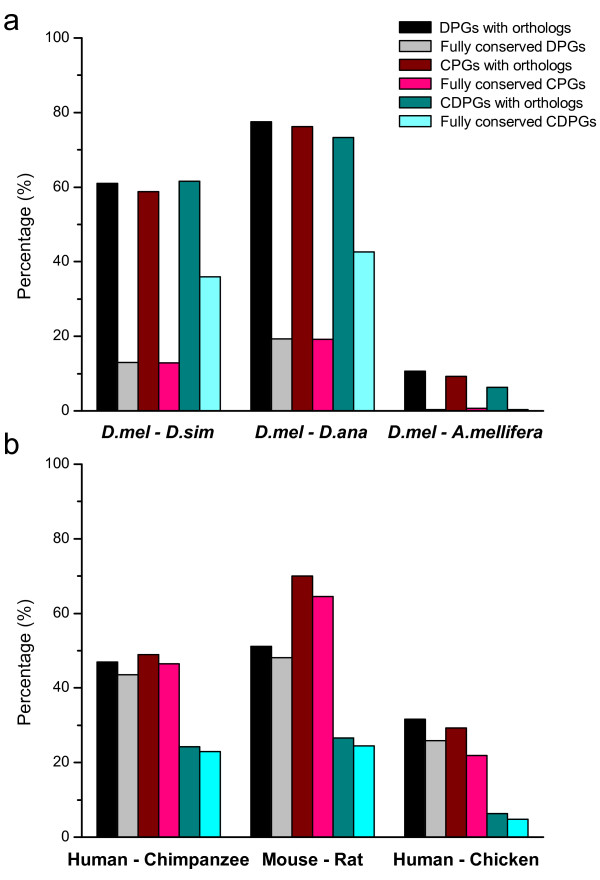
**Conservation of DPGs, CPGs, and CDPGs**. The conservation of DPGs, CPGs, and CDPGs are compared among insect (a) and vertebrate (b) genomes. A detailed classification for these genes is described in Material and Methods.

Although the conservation of gene organization in an evolutionary context must have functional relevance [28,29], we are not convinced that the degree of conservation is universal among different animal lineages. We selected three groups of species pairs with comparable divergence time to examine the difference in the divergent organization between insects and vertebrates [30-32], and defined DPGs into five different groups based on their degrees of evolutionary conservation [see Additional file 5 and Methods]. First, among the insect genomes studied, the gene pairs in the categories of DPGs with orthologs, convergently-paired genes (or CPGs) with orthologs, and co-directionally-paired genes (or CDPGs) with orthologs are all abundant and at the same magnitude as compared to the corresponding fully-conserved category of gene pairs in insects (Figure 3a) and vertebrates (Figure 3b). Second, the proportions of fully-conserved DPGs, CPGs, and CDPGs are all present at lower level as compared to those gene pairs with orthologs. The reduced abundance suggests relatively poorer conservation and greater dynamics, especially when their functional relevance is considered. Third, among the paired genes with orthologs, there are more than twice as many CDPGs as DPGs and CPGs, and CDPGs seem better conserved than the other two categories among insects. Fourth, among the vertebrate genomes analyzed, the fully-conserved DPGs, CPGs, and CDPGs remain at the same magnitude as the paired genes with orthologs, in sharp contrast with those found in insects. This observation suggests that the fully-conserved vertebrate DPGs are more conservative than those of insects. Fifth, among the vertebrates, CDPGs are much less abundant, less than half of the other two groups of genes, DPGs and CPGs (Figure 3b).

To investigate when the enrichment of DPGs was evolved among different species, we selected *Dmel *and human as models for a comparative analysis with regards to the short-term and long-term evolutions. There is little difference in the numbers of fully-conserved DPGs between *Dmel *and other *Drosophila *genomes (Table 1). However, there is a clear divide in DPGs between vertebrates and other eukaryotes; there are significantly more fully-conserved DPGs in the vertebrate lineage, especially among mammals (Table 2). The result suggested that most of the human DPGs might arise after the divergence of deuterostomes and protostomes. Alternatively, the conserved organizational features in the vertebrate lineage indicated that mechanistic differences might have evolved in the vertebrate lineage whereas DPGs in insects as well as the other two gene organizational structures, CPGs and CDPGs, are more dynamic or relatively less conserved. We also compared the percentage of DPGs with that of the randomly gene pairs in the fully-conserved category. We found that the occurrence of DPGs is significantly higher than that of the control in both insect (*p*-value = 3.81e-3, Fisher's Exact Test) and human genomes (*p*-value = 4.62e-4, Fisher's Exact Test). The result indicated that the generation of DPGs is not due to random events but selected along species evolution.

**Table 1:** Evolutionary conservation of DPGs in *D. melanogaster *as compared with those from other *Drosophila *species

	Fully Conserved	Both Orthologs	Species-specific	One Ortholog	No Ortholog
*D. simulans*	285	1057	422	243	219
*D. sechellia*	351	1108	382	216	169
*D. yakuba*	412	1197	246	155	216
*D. erecta*	487	1370	166	105	98
*D. ananassae*	425	1279	242	154	126
*D. pseudoobscura*	350	1215	316	175	170
*D. mojavensis*	317	1236	296	216	161
*D. virilis*	305	1258	301	204	158
*D. grimshwawi*	262	1143	327	264	230

**Table 2:** Evolutionary conservation of human DPGs as compared with those of other eukaryotic genomes

	Fully Conserved	Both Orthologs	Species-specific	One Ortholog	No Ortholog
*P. troglodytes*	622	47	430	163	165
*M. musculus*	708	22	319	225	153
*R. norvegicus*	606	35	328	255	203
*C. familiaris*	639	15	314	263	196
*G. gallus*	416	35	358	209	409
*D. melanogaster*	12	176	363	205	671
*C. elegans*	9	97	240	251	830
*S. cerevisiae*	0	18	153	141	1115
*S. pombe*	0	20	194	137	1076
*E. gossypii*	0	15	144	128	1140
*K. lactis*	2	15	163	118	1129
*M. grisea*	3	27	186	104	1107
*N. crassa*	0	27	210	100	1090
*A. thaliana*	3	91	233	205	895
*O. sativa*	0	28	131	200	1068

### DPGs are under stronger purifying selection

The conservation of gene organization could be explained by purifying selection (or negative selection) that refers to selection against nonsynonymous substitutions of protein-coding sequences. In this case, the evolutionary distance based on synonymous substitutions is expected to be greater than the distance based on nonsynonymous substitutions. We evaluated the rate s of nucleotide substitutions at synonymous (*Ks*) and nonsynonymous (*Ka*) sites for orthologs of both DPGs and non-divergently paired genes between *Dmel *and *D. pseudoobscura *for insects (Table 3) and between human and mouse for vertebrates (Table 4). In insects, the nonsynonymous substitution rate, *Ka*, is very similar between DPGs and the non-divergent gene sets. However, there is a significant difference in the synonymous substitution rate: the *Ks *values for DPGs are greater than those for the non-divergent genes. Furthermore, *Ka*/*Ks *ratios for DPGs are also significantly less than those for the non-divergent genes. In vertebrates, the *Ka *and *Ks *values for DPGs are both significantly less than those for the non-divergent genes, and the result implies that there are much fewer DNA substitutions happened in DPGs than in the non-divergent genes. Moreover, *Ka*/*Ks *ratios for DPGs are also significantly less than those for the non-divergent genes. These results suggested that DPGs are subjected to greater purifying selection than non-divergent genes. Although the negative selection of DPGs may not be directly attributable to the maintenance of their organizational characteristics, it represents a collective effect of both structural and functional importance.

**Table 3:** *Ka *and *Ks *for divergent and non-divergent *D. melanogaster *genes

	*Ka*^a^	*p*-value^b^	*Ks*^a^	*p*-value^b^	*Ka/Ks*^a^	*p*-value^b^
DPGs	0.087 ± 0.102	1.374e-3	1.582 ± 1.039	1.206e-10	0.074 ± 0.188	0.006
Non-divergent Genes	0.086 ± 0.103	0.135	1.467 ± 1.027	3.120e-4	0.085 ± 0.314	0.532
All Genes	0.087 ± 0.103		1.504 ± 1.032		0.081 ± 0.280	

^a ^Mean ± standard deviations.

^b ^*p*-value by Kolmogorov-Smirnov Test.

**Table 4:** *Ka *and *Ks *for divergent and non-divergent human genes

	*Ka*^a^	*p*-value^b^	*Ks*^a^	*p*-value^b^	*Ka/Ks*^a^	*p*-value^b^
Divergent Genes	0.098 ± 0.139	< 1e-10	0.449 ± 0.148	< 1e-10	0.201 ± 0.169	< 1e-10
Non-divergent Genes	0.244 ± 0.277	7.124e-3	0.776 ± 0.582	8.473e-3	0.281 ± 0.218	0.358
All Genes	0.234 ± 0.273		0.754 ± 0.569		0.276 ± 0.216	

^a ^Mean ± standard deviations.

^b ^*p*-value based on Kolmogorov-Smirnov Test.

### Co-expression of DPGs in *D. melanogaster*

**Figure 4:**
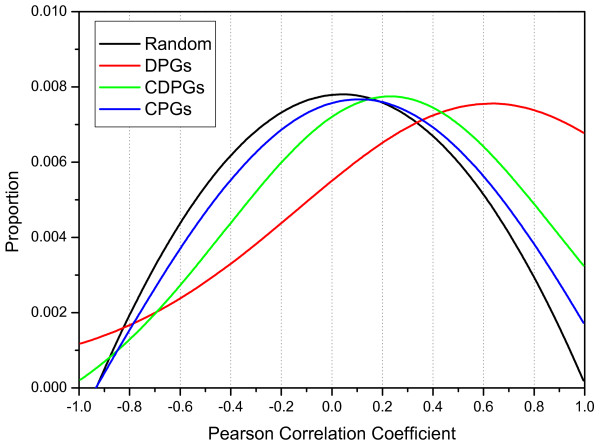
**The expression correlation analysis for DPGs**. The distributions of DPGs, CDPGs, CPGs, and random gene pairs are depicted in red, green, blue, and black, respectively. Each distribution is averaged over three microarray datasets. DPGs show stronger positive correlation in gene expression than CDPGs, CPGs, and random gene pairs.

It has long been known that transcriptional regulation is related to chromosomal structures and epigenetic controls. Neighbouring gene pairs are more likely to co-express than random gene pairs [3-7]. Furthermore, DPGs showed significant expression correlation than other types of consecutive gene pairs in human [10], fruit fly [12], and prokaryotes [33]. To test this hypothesis, we related DPGs to three microarray datasets for expression analysis (Methods). We mapped 351, 381, and 1,761 gene pairs with available microarray data in DeGregorio2001, Arbeitman2002, and Spellman2002 dataset, respectively. We calculated the Pearson correlation coefficient for all DPGs for each dataset independently, and found that the expression of DPGs is positively correlated better than CDPGs, CPGs, and random gene pairs in all three microarray datasets (Figure 4).

We also evaluated the significance of each correlation for all datasets. We denoted a correlation as a significant correlation when its *p*-value < 0.05, in which a significant positive correlation if the correlation is positive, otherwise a significant negative correlation. Of total 1,770 DPGs with available microarray data, 1,031 (58.2%) and 404 (22.8%) pairs showed significant positive and negative correlations respectively, which have *p *< 0.05 at least in one dataset. Moreover, there were 67 (3.8%) pairs showed either significant positive or negative correlations depending on conditions of microarray experiments [see Additional file 6].

The fact that the overall 84.8% of all DPGs are significant correlated with expression implies co-regulation as the driving force for maintaining this gene organization. In addition, the relationship between the intergenic distance of DPGs and the level of co-expression are not correlated as shown previously in human [10].

### Functional classification of DPGs

Previous studies have shown that many DNA repair genes are DPGs in human genome [8,10]. To observe the functional classification of DPGs in *Dmel*, we analyzed their annotations and included six other eukaryotic genomes, *H. sapiens*, *M. musculus*, *G. gallus*, *C. elegans*, *S. cerevisiae*, and *A. thaliana *for comparison [see Additional file 7]. In Biological Process, the GO terms related to organization/biogenesis and metabolic/biosynthetic processes topped the list of DPGs. The GO terms involved in RNA Binding are significantly overrepresented as compared to others in Molecular Function. From the results of Cellular Component, we found that DPGs tend to be in the classes of Intracellular, Organelle, Cytoplasm, and Protein Complex. However, almost 80% of the overrepresented GO terms in human and mouse are identical due to their close evolutionary distance. Above 90% of the terms in chicken are also present in human genome, but the total number of the overrepresented GO terms is less than human partly because of the rarity of GO annotations and less number of genes in the chicken genome. The *Dmel *DPGs had the most overrepresented GO terms among the eukaryotes accounted for the highest proportion of DPGs relative to gene density. Almost all terms found in human genome are also present in *Dmel *genome. Furthermore, every species has its own specific GO annotations, suggesting that some DPGs of different species may evolve independently during evolution. For instance, *C. elegans *has distinct overrepresented GO terms in Biological Process, including Reproduction, Behaviour, Growth and Development. The terms Thylakoid, Plastid, and Triplet Codon-Amino Acid Adaptor Activity in *A. thaliana *represent characteristics of plants that are different from those highlighted among animals. In *Dmel*, there were relatively more specific GO terms than other eukaryotes, including Transport, Cytoskeleton Organization and Biogenesis, Cell Death and Cell Proliferation in Biological Process, Nuclear Envelope, Cytoskeleton and Cytoplasmic Membrane-bound Vesicle in Cellular Component, Chromatin Binding, Motor Activity, Actin Binding, Kinase Activity, Cytoskeletal Protein Binding, Enzyme Regulator Activity, and Transcription Regulator Activity in Molecular Function.

**Figure 5:**
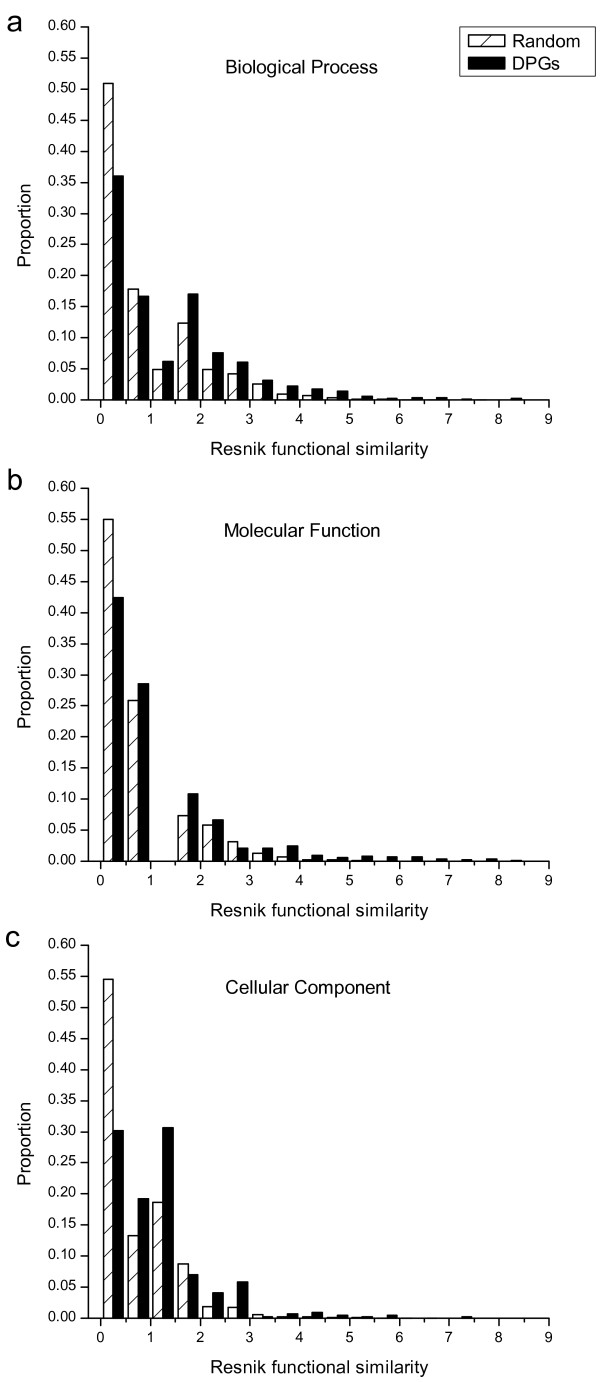
**The distribution of Resnik functional similarity for DPGs**. The statistics consists of GO subsystems "Biological Process", "Molecular Function" and "Cellular Component". The shaded and solid bars depict 50,000 random gene pairs and DPGs in *Dmel*, respectively.

We evaluated functional similarities for annotated *Dmel *DPGs using the Resnik semantic measure [34]. The functional similarities of DPGs were significantly larger than random gene pairs confirmed with Kolmogorov-Smirnov test (Figure 5). The *p*-values of these tests are 5.92e-12 for Molecular Function and less than 1e-16 for Biological Process and Cellular Component. These results indicated that the functions of DPGs are significantly different from random gene pairs and strongly biased toward functional similarities.

It has been reported that among prokaryotes there is a strong enrichment of DPGs in which one gene encodes a transcriptional regulator (R) and the other encodes other protein classes (X) [33]. This suggests potential regulatory interactions among DPGs. For the *Dmel *DPGs, we identified 459 (21.8%), 39 (1.9%), and 1,607 (76.3%) pairs for RX, RR, and XX structures, respectively. However, in the control set with 50,000 random gene pairs, we found 9,350 (18.7%), 500 (1%) and 40,150 (80.3%) pairs for RX, RR, and XX structures, respectively. Since *p*-values based on Fisher's Exact Test are 4.06e-3 for RX, 6.04e-4 for RR, and 0.138 for XX, RX and RR structures are more likely to present in DPGs. Of the 459 DPGs with RX structure, 318 pairs (69.3%) are fully-conserved across at least seven *Drosophila *clades. Furthermore, as a fraction of DPGs classified as XX may in fact play role as post-transcriptional regulators and some poorly annotated ('hypothetical') genes classified as X, RX structure should be more enriched in DPGs.

### Analysis of the promoter sequences among DPGs

**Figure 6:**
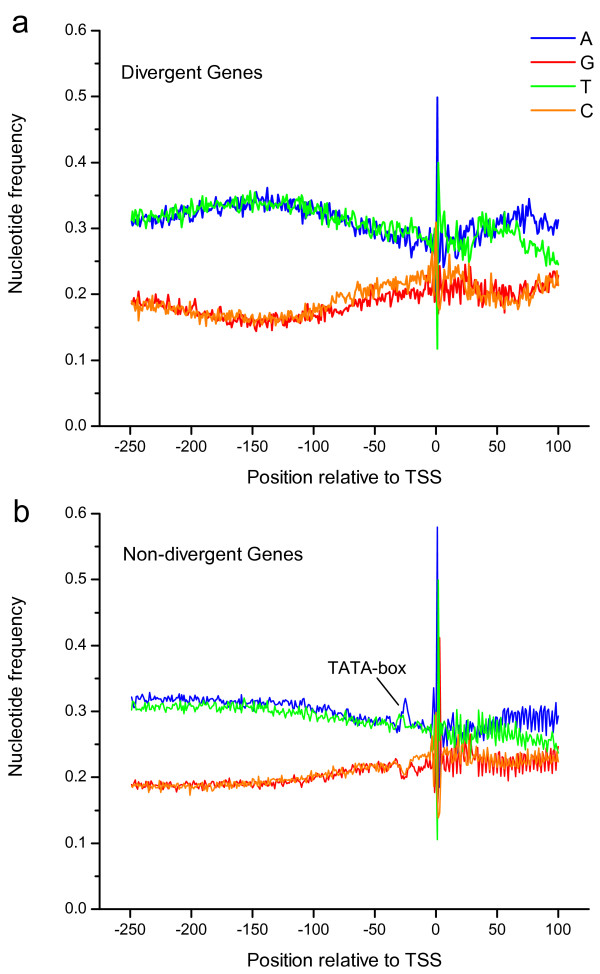
**Nucleotide frequencies around transcriptional start sites**. The statistics of nucleotide frequencies around transcriptional start sites (TSSs) for divergent (a) and non-divergent (b) genes in *Dmel *are plotted. The X-axis shows the positions relative to TSS between -250 bp and 100 bp. There is a distinct TATA-box in non-divergent gene promoters.

Previous studies on human genome have shown evidence that the majority of RNA polymerase II-transcribed genes with bidirectional promoters have a CpG island between them[8] and the promoters of DPGs have a higher median GC-content than non-divergent promoters [10]. For *Dmel*, the median GC-content of divergent promoters is37%, significantly less than the median value 50% of non-divergent promoters (Welch Two-Sample T-test, *p*-value < 1e-16). The contradictory result mainly stemmed from the genome GC-content difference of the two species. T he majority of mammalian promoters are associated with CpG islands that do not exist in many other species because of the absence of DNA methylation, including *Drosophila*. We evaluated the average nucleotide composition around the TSSs of divergent and non-divergent genes in *Dmel*. The nucleotide frequency of non-divergent genes demonstrated the presence of TATA-box and absence of DPGs (Figure 6). It is consistent with the result from an analysis on human DPGs[10].

There are four core promoter elements that have been experimentally identified among *Drosophila *promoters: TATA box, Initiator (Inr), Downstream Promoter Element (DPE), and Motif Ten Element (MTE) [35,36]. We identified 1,755 and 4,623 genes with at least one count of the four core promoter elements in DPGs and non-divergent genes, respectively (Table 5). According to Fisher's Exact Test, we learnt that TATA-box, DPE, and MTE are significantly less than expected at a cut-off of *p *< 0.05, but there is not a single core promoter element dominating in DPGs. As core promoter elements usually work in cooperation, we chose to analyze combinations of core promoter elements utilized by DPGs, and found that the Inr-DPE pair showed significantly greater value than the expected (*p*-value = 3.95e-3, Fisher's Exact Test; Table 6).

**Table 5:** Core promoter elements in divergent and non-divergent *D. melanogaster *genes

	Total^a^	TATA	Inr	DPE	MTE
Divergent Genes	1628 (27.2%^c^)	227 (21.7%)	1040 (26.3%)	517 (23.5%)	158 (23.5%)
Non-divergent Genes	4361	819	2912	1688	515
*p*-value^b^		1.13e-6	0.34	6.07e-4	0.039

^a ^The number of genes containing at least one of the four core promoter elements.

^b ^*p*-value based on Fisher's Exact Test.

^c ^Percentage of DPGs with the core promoter elements.

**Table 6:** Combinations of core promoter elements in divergent and non-divergent genes of *D. melanogaster*

	Total^a^	TATA-Inr	Inr-DPE	TATA-DPE	TATA-MTE	Inr-MTE	DPE-MTE
DPGs	290 (17.8%^c^)	80 (14.6%)	181 (21.7%)	23 (11.6%)	12 (14.8%)	43 (15.4%)	20 (15.0%)
Non-divergent Genes	1337	469	653	176	69	236	113
*p*-value^b^		0.088	0.023	0.028	0.5533	0.3489	0.4782

^a ^The number of genes containing at least two of the four core promoter elements.

^b ^*p*-value based on Fisher's Exact Test.

^c ^Percentage of DPGs with core promoter elements.

## Discussion

### The limitation in defining DPGs based on shared distance

We determined DPGs based on the criterion that the two transcriptional start sites should be found in the opposite orientation and less than 1,000 bp apart so the gene pair has a greater possibility to be functionally correlated by sharing a common regulatory region. However, a recent study on human *CYP1A1 *and *CYP1A2 *genes [37] has shown that they are simultaneously controlled through bidirectional and common regulatory elements, but separated by 23 kb intergenic spacer region, suggesting that the number of DPGs are underestimated by current standard. Obviously, some of the true DPGs that are distantly situated are to be mapped experimentally in the future, perhaps coupled with the next-generation sequencing technology. Fortunately, the abundance of DPGs overcomes this obvious limitation for characteristic analysis unless individual genes are scrutinized.

### The conservation and origin of divergent gene organization

We found that the human DPGs and their corresponding orthologs are conserved only among vertebrates, especially among mammals. Similarly, the DPG orthologs of other species, such as those of insects, are also better conserved among their close relatives as compared to random genes. These observations support the idea that DPGs provide structural advantages for co-regulation so they become conserved when functionally important genes (such as certain housekeeping genes) become divergently organized. This hypothesis is further supported by the abundance of species-specific DPGs observed. For instance, human DPGs have strong association with CpG islands that are specifically related to genome compositional dynamics and evolution of mammalian genomes. In addition, some of the DPGs may be associated with species specific functions as DPGs in *C. elegans *are significantly associated with reproduction, behaviour, growth, and development related functions. The GO terms Thylakoid, Plastid, and Triplet Codon-Amino Acid Adaptor Activity in *A. thaliana *represent the characteristics of plants different from that of animals.

The conservation patterns of the divergent gene organization differ among different lineages, such as between insects and vertebrates. A majority of DPGs with orthologs of insect genomes are diminishing faster over evolutionary time scales but not those of vertebrates. We believe that the organizational priorities for the insect and vertebrate lineages are different strategically as insect species tend to be more diversified to adapt different environments and ecological relationships when compared to vertebrates that gain complex in terms of anatomical structures and behaviours over time.

The origin of DPGs has been of great interest since more and more individual divergent gene pairs have been identified experimentally and several hypotheses have been proposed to illustrate the molecular mechanisms as to how the architecture is created. The first argues for tandem duplication. Tandem duplicated genes refer to two gene s reside physically adjacent to each other, often in the same orientation, and usually have similar expression patterns and similar in function, if not identical. Tandem duplication is a common event among eukaryotic genomes, which is a primary mechanism for generating gene clusters. Genome analysis suggests that one copy of a duplicated gene could drift and potentially acquire a new function. For example, several odorant-binding proteins in *Drosophila *are transcribed in opposite directions [38]. Nevertheless, our analysis showed that only 4.3% of all the divergent gene pairs were tandem duplicates, suggesting that tandem duplication might not be the essential driving force. The second hypothesis is overprinting, which is a process of generating new genes from pre-existing nucleotide sequences [39]. For a divergent gene pair, one is often confined to a single lineage, but the other is widespread. For example, the two human genes *SIRT3 *and *PSMD13*, are linked in divergent configuration sharing regulatory mechanism [40]. One of them, *SIRT3*, only has orthologs in vertebrates, and the other, *PSMD13*, is found in all genomes examined in this study. It is assumed that the evolution of their divergent arrangements is associated with that of a complex pathway of co-regulation related to aging in vertebrates. Thus, *PSMD13 *represents an old gene widespread among eukaryotes but *SIRT3*is a vertebrate invention. The third theory is genome rearrangement. A genome rearrangement occurs when a chromosome breaks at two or more locations and reassembled in to a different orientation. This results in a DNA sequence that has essentially the same features as the original sequence, except that the order of these features has been modified. A possible scenario to explain the origin of DPGs is that this gene organization originated by chance via genome rearrangement. When a genome rearrangement brought two distant genes together and formed a divergent gene pair, there was no distinct functional relationship at the beginning. However, the promoter region shared by both genes was maintained by evolutionary pressure. A mutation in this region would be potentially hazardous, resulting in failure in normal expression for both genes. Both genes may have housekeeping roles and any one of them would be vital to survival. In the process of evolution, genome took advantage of such a gene organization and utilized it for transcriptional regulation. Regulation of gene expression by forming DPGs may result in more efficient control an d reduce the need for more complex regulatory pathways.

### The features of DSPs in eukaryotes

In general, the transcription of each gene in a eukaryotic genome is controlled independently and operons are unusual in eukaryotes, as opposed to most prokaryotes [41]. The organization of DSPs in DPGs does not exhibit universal structural features, because there have not been consistent sequence motifs found among them. As far as we know, promoters recognized by RNA polymerase II are divided into two broad categories: TATA-containing and TATA-less promoters. Tissue-specific genes typically contain TATA boxes located ~30-bp upstream of a single TSS.

Tissue-specific transcriptional factors generally bind upstream of a TATA box and either activate or repress promoter activities. The promoters of housekeeping genes do not generally contain TATA box sequences and usually display multiple transcription start sites. Housekeeping promoters are active in most cell types and often contain binding sites for ubiquitous transcription factors. Many of the human DSPs that have been studied so far are TATA-less [10] and associated with genes for housekeeping functions. Examples include DSPs of the genes encoding *DHFR/Rep-1 *[42], *TK/KF *[20], *Surf1/Surf2 *[43], *GPAT/AIRC*[22], histones *H2A/H2B*[21] and *BRCA1/NBR2 *[16]. Although a few DSPs have TATA boxes in both orientations[14], most lack TATA boxes and initiator elements in either direction and stimulate transcriptional initiation at multiple sites over broad initiation windows as a strong association between DPGs and CpG island is described in human genome [8,10]. The mammalian DSPs with CpG island are frequently lack of TATA boxes [44-46]. Nevertheless, CpG islands appear less frequently found in promoters that contain both TATA boxes and initiator regions [47]. It is clear that a majority of DPGs in human are co-regulated by TATA-less promoters with CpG-islands and Sp1 binding site is prevalent in DSPs [36]. Furthermore, some other transcriptional factor binding sites may also play key roles in regulating certain DPGs. Examples include:(1) YY1 factor binding site in *Surf1/Surf2 *genes [48,49], (2) CCAAT box binding sites for *HSF-1 *(Heat shock factor-1)/*Bop1*[50], *E14/ATM*[15], *BRCA1/NBR2*[16], and *GPAT/AIRC *[22], (3)GC boxes between the TSSs of *TAP1/LMP2*[51], *DHFR/Rep-1 *[42]and *GPAT/AIRC*[22], and (4)E2F factor binding site in *TK/KF *genes [20].

Our analysis for *Dmel *genome indicated that DPGs often have TATA-less promoters, consistent with the finding in human. However, CpG island is not an indicator for DSPs in *Dmel *as DNA methylation is known to be absent in this organism. In this study, we focused on four common core promoter elements experimentally identified: TATA box, Inr, DPE, and MTE. TATA box and Inr are well-known in Drosophila and vertebrates. The core motif of DPE is located exactly from +28 to +33 bp downstream of TSS and is recognized by two distinct TBP-associated factors (TAFs). Experimental evidence suggests that DPE appears to be as widely used as TATA box [52]. MTE is located at positions from +17 to +22 bp, experimentally verified to interact with TFIID [53]. These core promoter elements show organism-specific patterns; Inr has higher information content, and DPE is much more frequently found in the fly promoters as compared to those among mammals [54]. The diversity of core promoters are thought to contribute to specificity of gene regulation in a combinatorial fashion [55]. Although the distribution of the four core promoter elements indicated that there has not been a single element overrepresented among DPGs, a significant overrepresentation was found in a combination of Inr and DPE, which is functionally equivalent to CpG islands in mammalian DPGs.

## Conclusion

DPGs exist as one of the common structural features of genomes and provide advantages in transcriptional co-regulation. DPGs are abundant among eukaryotic genomes and highly conserved; the conservation is stronger within lineages than between lineages. The conservation patterns among the different organizational classes, i.e. DPGs, CPGs, and CDPGs, appear linage-specific as vertebrate DPGs are better conserved than those of insects. Further analyses revealed that DPGs are strongly co-regulated in expression profiles and associated with certain functional categories. DPGs are mostly housekeeping genes so they lack TATA box. Combinations of transcriptional factor binding sites are crucial in regulating this divergent gene organization.

## Methods

### Genomic data

We retrieved the genomic data and annotations for *D. melanogaster *and other nine *Drosophila *(*D. simulans*, *D. sechellia*, *D. yakuba*, *D. erecta*, *D. ananassae*, *D. pseudoobscura*, *D. mojavensis*, *D. virilis *and *D. grimshwawi*) from *Drosophila *Comparative Annotation (available at ) that host gene models built with Gene Wise based on Flybase Release 4.2 for *Dmel*. Other genome data from sixteen eukaryotes were downloaded from the NCBI Map Viewer , which include *Homo sapiens, Pan troglodytes, Mus musculus, Rattus norvegicus, Canis familiaris, Gallus gallus, Apis mellifera, Caenorhabditis elegans, Saccharomyces cerevisiae, Schizosaccharomyces pombe, Eremothecium gossypii, Kluyveromyces lactis, Magnaporthe grisea, Neurospora crassa, Arabidopsis thaliana, and Oryza sativa*. The genome sizes of all analyzed species were obtained from NCBI .

### Identification of DPGs

Divergently-paired genes or DPGs are defined as divergently-arranged (bi-directional or head-to-head) gene pairs on opposite strands with transcription start sites within 1,000 bp [10]. We did an all-against-all BLAST search on all *Dmel *genes. The tandem duplicates were determined as neighbouring gene pairs with expect value E < 1e-10. The definitions of CPGs and CDPGs are associated to that of DPGs, where TSS distances are within 1 kb.

### Organizationally-conserved DPGs between *Dmel *and other species

The orthologs among *Dmel *and other *Drosophila *clades as well as from other eukaryotic genomes were extracted in a similar way as we did for *Dmel *from *Drosophila *Comparative Annotation and NCBI HomoloGene release 56 . According to the degree of conservation, we classified DPGs into five categories [see Additional file 5]. (1) "Fully conserved" are DPGs that have orthologs for both genes and remain their relative directions in other species. (2) "Both orthologs" represents DPGs that have orthologs for both genes but lost the divergent relationship in other species. (3) "Species-specific" is defined as those that have one ortholog in other species but chose another gene without orthology as a counterpart to keep their relative direction. (4) "Single ortholog" means DPGs that only have one gene ortholog found in other species but lost the counterpart. (5) "No ortholog" refers DPGs that do not have orthologs in any other species analyzed. We also prepared 20,000 gene pairs randomly selected separately from the *Dmel *and human genomes in order to show the evolutionary conservation among DPGs.

We selected three groups of species pairs with comparable divergence time to examine the difference of divergent organization in the light of evolution in of insect and vertebrate lineages. The first group is composed of *D. melanogaster *vs. *D. simulans *and human vs. chimpanzee, which diverged about 5 million years ago. The second group includes *D. melanogaster *vs. *D. ananassae *and mouse vs. rat; both have a divergence time about 40 million years. The third group concerns *D. melanogaster *vs. *A. mellifera *and human vs. chicken; both have a relatively longer divergence time about 300 million years.

### Synonymous and nonsynonymous substitution rates of DPGs

We calculated synonymous (*Ks*) and nonsynonymous (*Ka*)substitution rates [56] for both divergent and non-divergent orthologous gene pairs between *Dmel *and *D. pseudoobscura *for insects and between human and mouse for vertebrates using a maximum likelihood (ML) algorithm that corrects for reversion events implemented in the software package PAML [57]. Protein identity was calculated based on multiple alignments by using CLUSTALW [58].

### Expression correlations among DPGs

The expression data based on microarray experiments belong to three datasets: DeGregorio2001, Arbeitman2002, and Spellman2002. The DeGregorio2001 dataset is from adult flies in response to microbial infection, and it was acquired from high-density oligonucleotide microarrays [59] representing 13,172 distinct genes and 351 DPGs. The Arbeitman2002 dataset was generated from a study on the development of *Dmel *measured in a time-course [60]; it contains 6,841 distinct genes and 381 DPGs. The Spellman2002 dataset has 13,141 distinct genes determined from over 80 experimental conditions [5] and contains 1,761 DPGs. We defined the level of co-expression between two genes as Pearson correlation coefficient of expression abundance, and denoted a significant correlation as *p*-value < 0.05. We also selected CDPGs, CPGs, and 20,000 random gene pairs as a control for each dataset to calculate correlation coefficient.

### Gene Ontology (GO) annotation for DPGs

To determine statistically overrepresented GO terms for DPGs, we counted the number of appearances of each GO term in general annotations for DPGs and all other genes. We used generic GO terms to offer a broad overview of the ontology content without details of specific terms. For each GO term, a *p*-value is calculated based on hypergeometric test to represent the probability that the observed number of gene counts within a GO group could have resulted from a random distribution between the tested and the reference groups. The statistically overrepresented GO terms or number of genes can be identified when *p*-value is less than 0.05 based on Benjamini & Hochberg False Discovery Rate (FDR) correction[61].

We evaluated the functional similarities between DPGs using the Resnik semantic measure [34]; this measure is based on the information content of shared parents of the two GO terms. We denoted *N*(*C*_*i*_) as the number of *Dmel *genes annotated by GO term *C*_*i*_, and the Resnik probability *p*(*C*_*i*_) is defined as *p*(*C*_*i*_) = *N*(*C*_*i*_)/*N*(*root*). In term of the directed acyclic graph (DAG) structure of Gene Ontology, this implies that *p*(*C*_*i*_) is monotonically non-decreasing as one moves up to root term: if *C*_*i *_is_a *C*_*j*_, then *p*(*C*_*i*_) ≤ *p*(*C*_*j*_). Moreover, a root term has a Resnik probability of 1, and a non-root term has a Resnik probability less than 1. If two genes *g*_1 _and *g*_2 _annotated by GO terms *C*_1 _and *C*_2_, respectively, the functional similarity between genes *g*_1 _and *g*_2 _is determined by Equation (1):

$$F_{sim}(g_{1},g_{2})=max_{C_{i}\in S(C_{1},C_{2})}\{-ln[p(C_{i})]\}$$

where *S*(*C*_1_, *C*_2_) is the set of general GO terms shared by both *C*_1 _and *C*_2_.

We also prepared 50,000 gene pairs randomly selected from the *Dmel *genome in order to show the functional relevance among DPGs. The difference between the distribution of DPGs and the control sets was analyzed by using Kolmogorov-Smirnov test to confirm whether DPGs are inclined to have similar function.

We identify a gene as a transcriptional regulator (R) if it is annotated with GO term "regulation of biological process" in the general annotation, whereas any other class of proteins (X)are treated separately. Gene pairs are classified into three classes: regulator-regulator pairs (RR), potential regulatory interactions (RX), and non-regulatory (XX). The 50,000 random gene pairs mentioned above are used as a control set.

### Analysis of promoter sequence

We extracted the sequence from -250 to +100 relative to TSS for all *Dmel *genes. There are four core promoter elements that have been experimentally identified in *Drosophila *promoters: TATA box, Initiator (Inr), Downstream Promoter Element (DPE), and Motif Ten Element (MTE) [35,36]. According to a recent study about the features of *Drosophila *core promoters [62], we identified these core promoter elements for all genes based on consensus sequences and functional integrity for each element (Table 7). Because these core promoter elements usually work in coordination, we also analyzed combinations of any two elements. Fisher's Exact Test was used to determine whether a core promoter element or a combination differed from the expected at a significance cut-off of *p*-value < 0.05.

**Table 7:** The parameters of core promoter elements

Name	Consensus^a^	Length/Center^b^	Window^c^	Mismatch allowed^d^
TATA box	TATAWAAR	12/3	-33 - -23	1
Initiator	TCAKTY	12/3	-1 – +9	1
DPE	RGWYV	8/0	+27 – +36	0
MTE	CSARCSSAAC	10/0	+17 – +26	2

^a ^Motif consensus in NC-IUB nomenclature

^b ^The length of motifs (left) and the distance between the center and 5' end (right)

^c ^Applied windows for the center of motifs

^d ^The maximal number of allowed mismatches for motif consensus to remain functional

## Abbreviations

*Dmel*: *Drosophila melanogaster*; DPG: divergently-paired gene; CPG: convergently-paired gene; CDPG: co-directionally-paired gene; TSS: transcription start site; DSP: divergently-shared promoter; Inr: Initiator; DPE: Downstream Promoter Element; MTE: Motif Ten Element.

## Authors' contributions

LY designed and performed data analysis and drafted the manuscript. JY designed and supervised the project and revised the manuscript. Both authors read and approved the manuscript.

## Supplementary Material

Additional file 1 **Table S1.** identification and characterization of DPGs in *Dmel* Click here for file

Additional file 2 **Table S2.** identification and characterization of DPGs in selected eukaryotes Click here for file

Additional file 3 **Figure S1.** the distributions of TSS distance of DPGs among selected eukaryotic genomes Click here for file

Additional file 4 **Table S3.** the distribution of DPGs among eukaryotic genomes Click here for file

Additional file 5 **Figure S2.** the classification of DPGs based on organizational conservations Click here for file

Additional file 6 **Table S4.** the significance of the expression correlation for DPGs in *Dmel* Click here for file

Additional file 7 Table S5. the significantly overrepresented GO terms in DPGs Click here for file
