# Supplementary material for: A comparative analysis of divergently-paired genes (DPGs) among Drosophila and vertebrate genomes
Source: BMC Evol Biol. 2009 Mar 11;9:55. doi: 10.1186/1471-2148-9-55 (PMC2670823; doi:10.1186/1471-2148-9-55)
Supplement: Additional file 3 — Figure S1. the distributions of TSS distance of DPGs among selected eukaryotic genomes [file 1471-2148-9-55-S3.pdf]

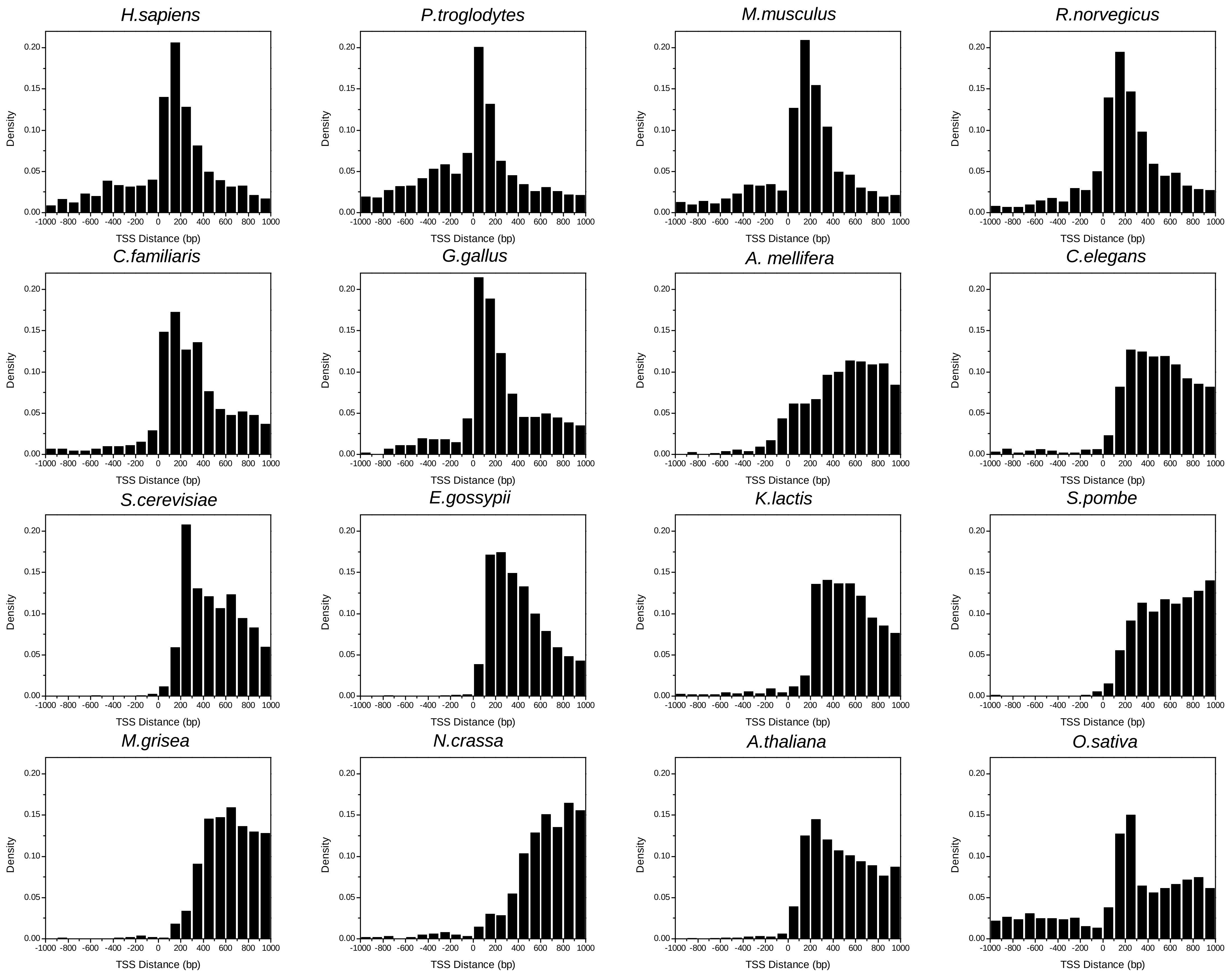

H.sapiens
P.troglodytes
M.musculus
R.norvegicus
C.familiaris
G.gallus
A. mellifera
C.elegans
S.cerevisiae
E.gossypii
K.lactis
S.pombe
M.grisea
N.crassa
A.thaliana
O.sativa
Density
0.00
0.05
0.10
0.15
0.20
TSS Distance (bp)
-1000 -800 -600 -400 -200 0 200 400 600 800 1000
