# Supplementary material for: A comparative analysis of divergently-paired genes (DPGs) among Drosophila and vertebrate genomes
Source: BMC Evol Biol. 2009 Mar 11;9:55. doi: 10.1186/1471-2148-9-55 (PMC2670823; doi:10.1186/1471-2148-9-55)

# Fully conserved

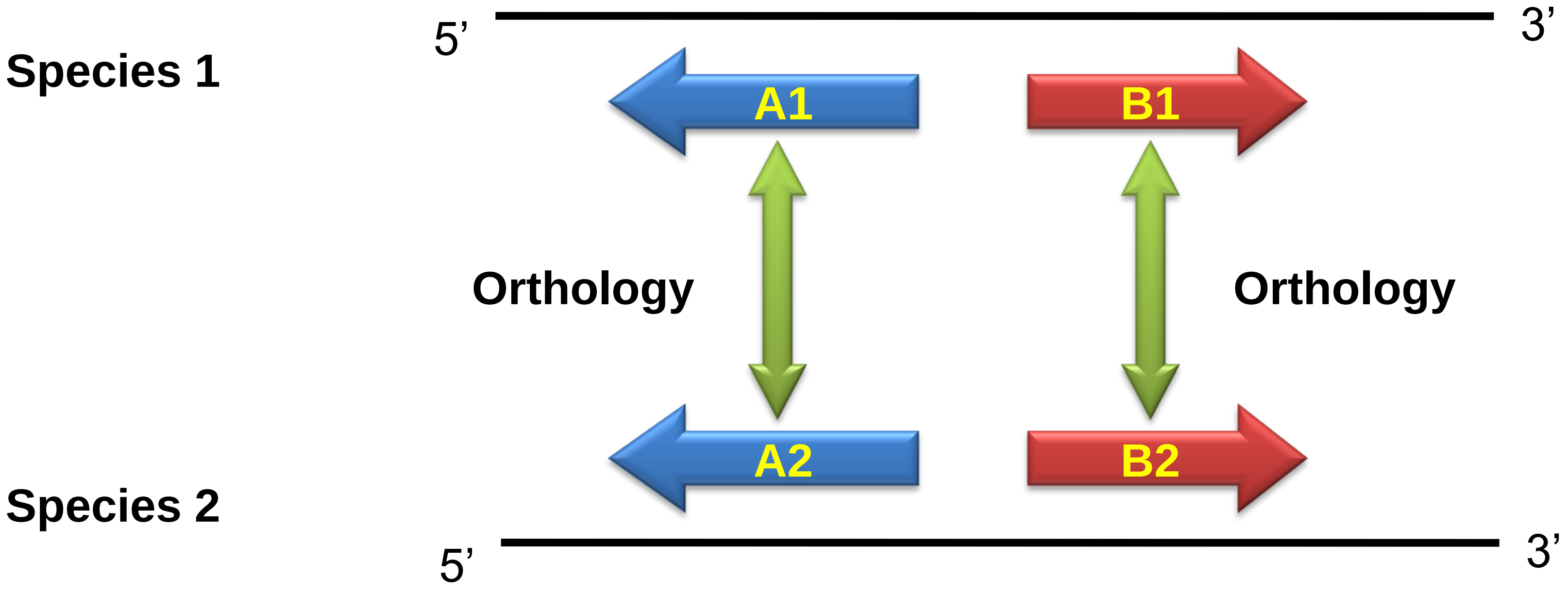

# Both orthologs

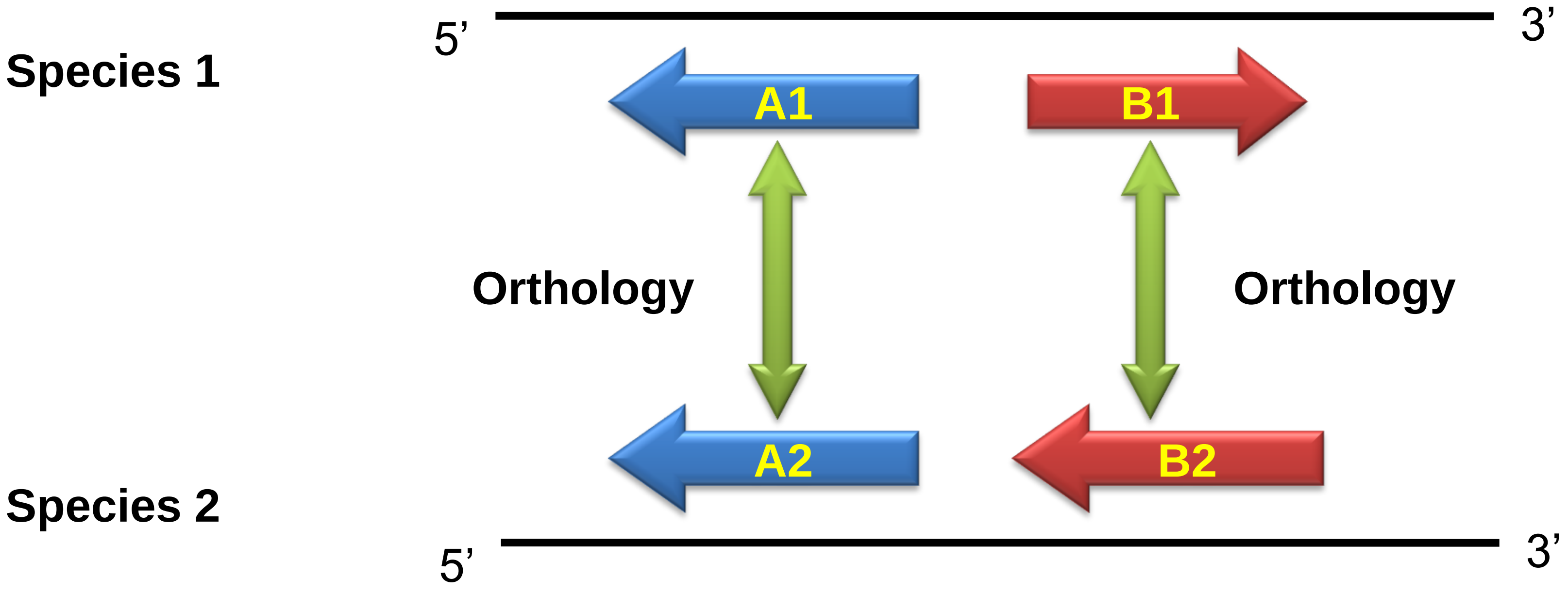

# Species-specific

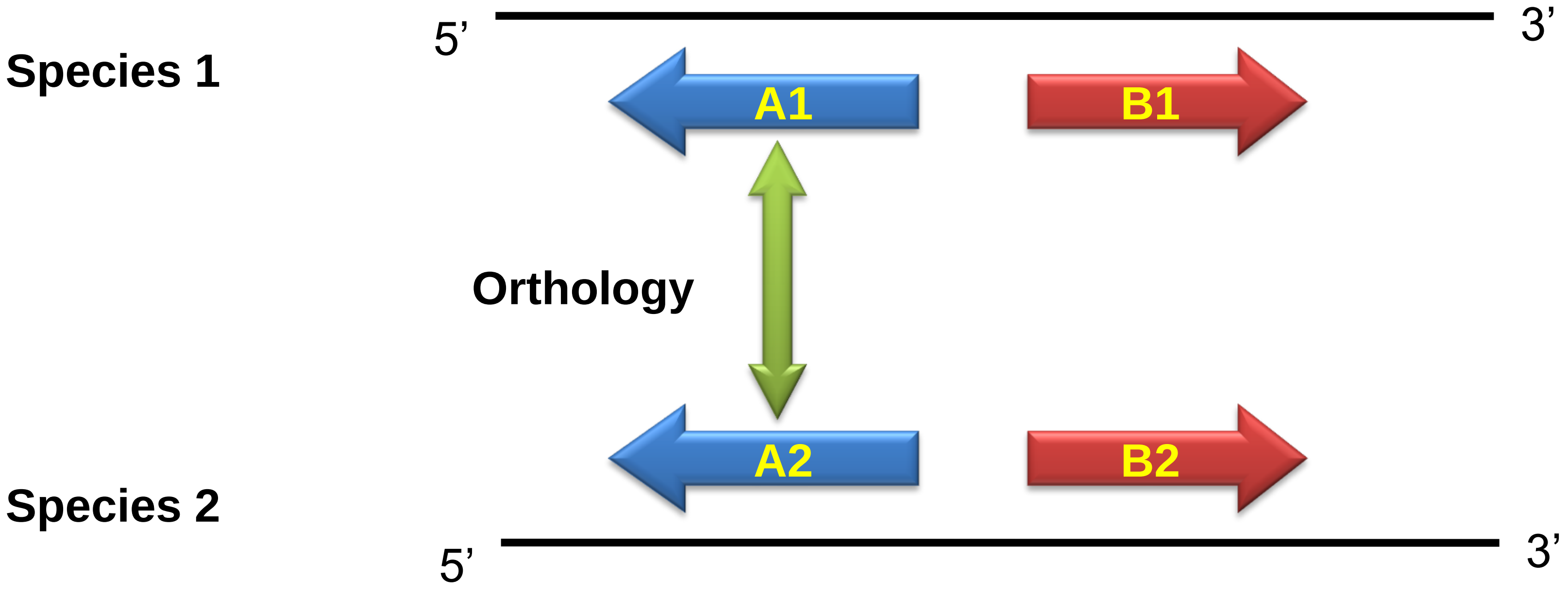

# Single ortholog

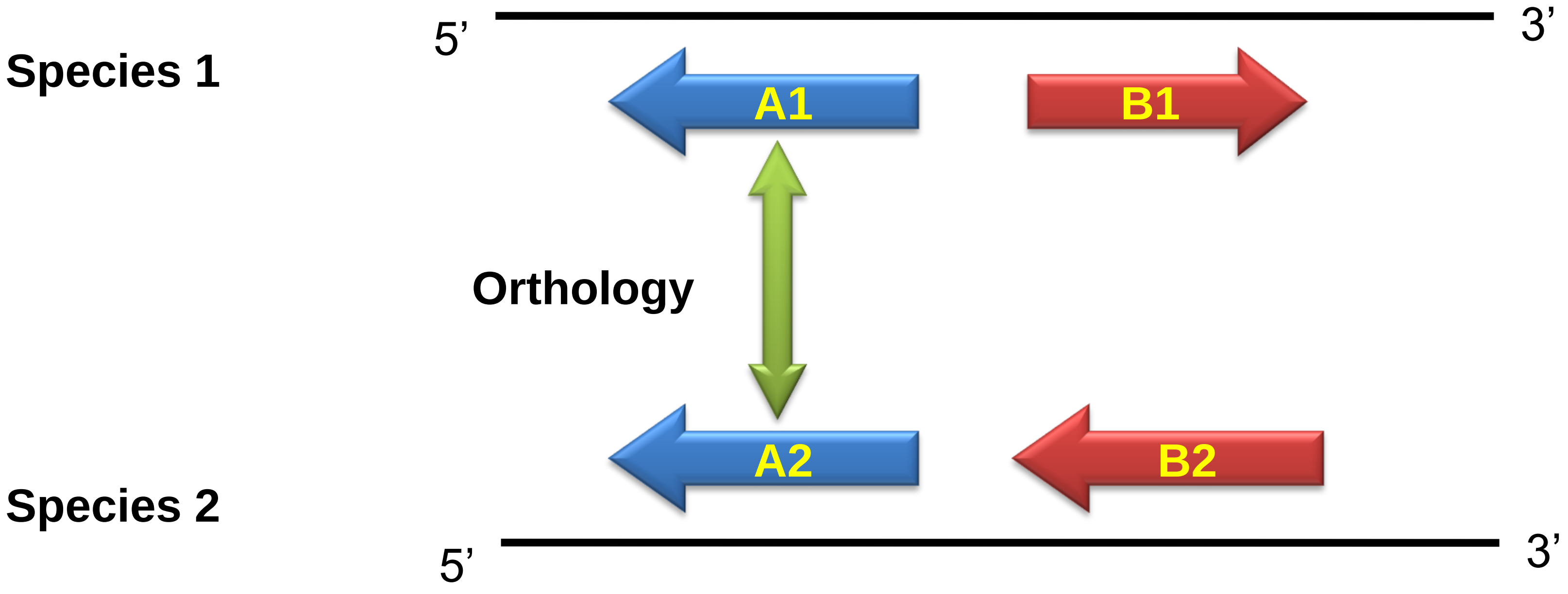

# No ortholog

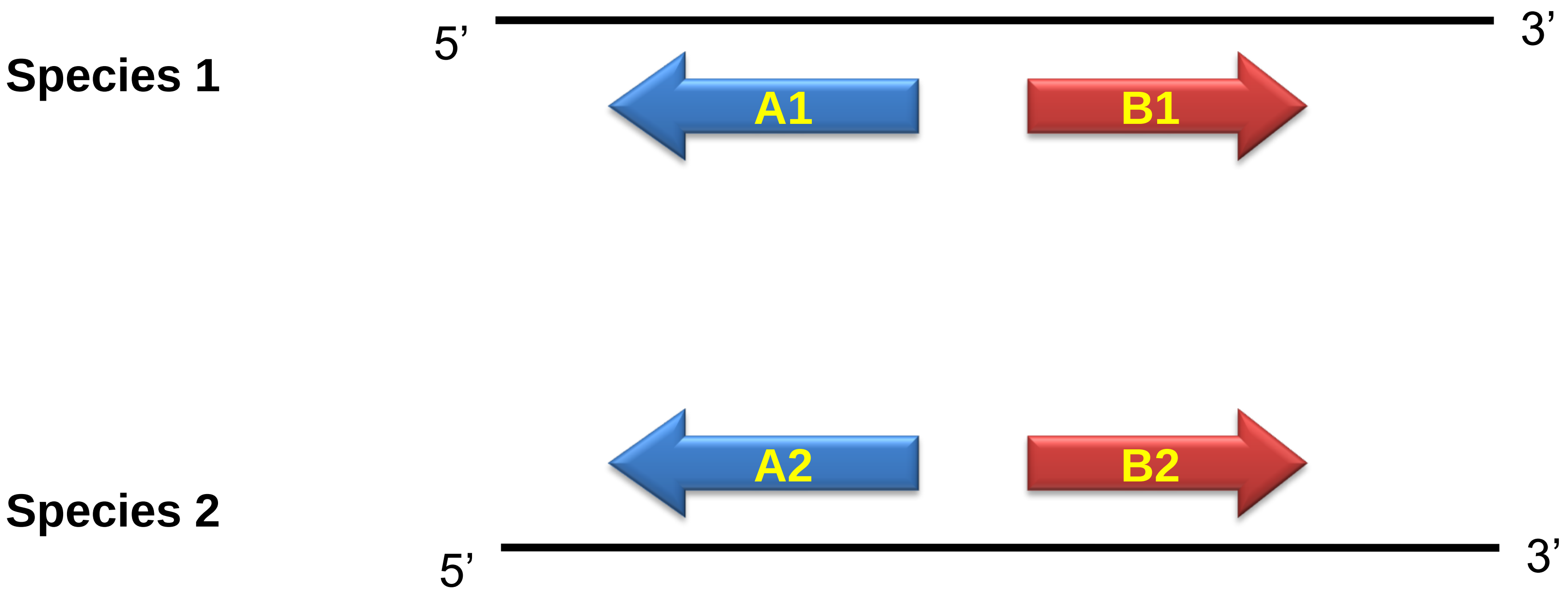

Supplement: Additional file 5 — Figure S2. the classification of DPGs based on organizational conservations [file 1471-2148-9-55-S5.pdf]
